# High throughput discovery of thermo-responsive materials using water contact angle measurements and time-of-flight secondary ion mass spectrometry

**DOI:** 10.1002/sia.4910

**Published:** 2012-03-08

**Authors:** Andrew L Hook, David J Scurr, Daniel G Anderson, Robert Langer, Paul Williams, Martyn Davies, Morgan Alexander

**Affiliations:** a Laboratory of Biophysics and Surface Analysis, School of Pharmacy, University of NottinghamNottingham, NG7 2RD, UK; b David H. Koch Institute for Integrative Cancer Research, Massachusetts Institute of Technology77 Massachusetts Avenue, Cambridge, MA 02139, USA; c School of Molecular Medical Sciences, Centre for Biomolecular Sciences, University of NottinghamNottingham, NG7 2RD, UK

**Keywords:** thermo-responsive, stimuli, switchable, ToF-SIMS, water contact angle, polymer microarray

## Abstract

Switchable materials that alter their chemical or physical properties in response to external stimuli allow for temporal control of material-biological interactions, thus, are of interest for many biomaterial applications. Our interest is the discovery of new materials suitable to the specific requirements of certain biological systems. A high throughput methodology has been developed to screen a library of polymers for thermo-responsiveness, which has resulted in the identification of novel switchable materials. To elucidate the mechanism by which the materials switch, time-of-flight secondary ion mass spectrometry has been employed to analyse the top 2 nm of the polymer samples at different temperatures. The surface enrichment of certain molecular fragments has been identified by time-of-flight secondary ion mass spectrometry analysis at different temperatures, suggesting an altered molecular conformation. In one example, a switch between an extended and collapsed conformation is inferred. Copyright © 2012 John Wiley & Sons, Ltd.

## Introduction

Controlled capture and release from surfaces of biomolecules and biomolecular assemblies, such as eukaryotic cells, has been the focus of numerous studies and has been achieved using thermo-responsive hydrogels such as poly(*N*-isopropyl acrylamide) (pNIPAM).^1^–^5^ This polymer has been extensively used to temporally control cell attachment by exploiting its transition between a swollen and collapsed state by altering the temperature above and below the lowest critical solution temperature.^1^ Alternatives to pNIPAM-based thermo-responsive hydrogels have been explored such as polymers containing the ethylene glycol moiety,^6^,^7^ for example, using 2-(2-methoxyethoxy)ethyl methacrylate (MEO_2_MA) and oligo(ethylene glycol) methacrylates.^8^–^10^ To enlarge the scope of biological and physical applications where switchable materials can be applied, a broadened library of thermo-responsive materials is of interest. Recently, polymer microarrays have become a key tool for the discovery of novel polymers.^11^–^13^ High throughput surface characterisation has also been developed on this platform and has enabled the elucidation of structure-function relationships.^11^,^14^–^17^ Recently, a study used polymer microarrays to screen for temperature-responsive materials based upon the thermal release of attached eukaryotic cells.^18^ We use a different approach to identify thermo-responsive materials, carrying out a direct screen of water contact angle (WCA) switching to identify thermo-responsive materials rather than implying switchability through cell detachment. High throughput WCA measurements were utilised to identify polymers with thermo-responsive properties from a library of 279 unique materials in a polymer microarray format. The surface sensitivity and molecular specificity of time-of-flight secondary ion mass spectrometry (ToF-SIMS) was exploited to investigate temperature-dependant conformational changes at the surface of the ‘hit’ polymers.^19^,^20^

## Experimental

### Polymer polymerisation

Polymer microarrays were formed using a XYZ3200 pin printing workstation (Biodot, Irvine, CA, USA) as described previously.^15^ Slotted metal pins (946MP8B, Arrayit, Sunnyvale, CA, USA) with a tip diameter of 295 µm were used to transfer approximately 4 nl of polymerisation solution onto poly(2-hydroxyethyl methacrylate) (pHEMA) dip-coated substrates^21^ before slides were irradiated with a long wave ultraviolet (UV) source for 1 min, resulting in an average polymer spot size of 435 µm. For formation of polymer coupons, 8 µl of polymerisation solution was dispensed in triplicate onto a pHEMA-coated substrate or onto a 1.5 × 1.5 cm silicon wafer for ToF-SIMS samples. Polymer coupons were polymerised in an argon atmosphere (O_2_ < 1300 ppm) by photopolymerisation with a long wave UV source for 10 min. Polymerisation solution was composed of 75% (v/v) monomer (Sigma, Dorset, UK), 24% (v/v) DMF and 1% (w/v) photoinitiator 2,2-dimethoxy-2-phenylacetophenone. Samples were subsequently dried at <50 mTorr for 7 days. The monomers are shown in Fig. SI1 (Supporting Information).

### Water contact angle measurements

Sessile WCA measurements were taken of each polymer as previously described.^16^ The temperature of an aluminium stage was regulated using an FBC 735 Temperature Controller (Fisherbrand, Loughborough, UK). Samples were held at a constant temperature for 30 min before WCA measurements were taken.

### Time-of-flight secondary ion mass spectrometry

The ToF-SIMS analysis was performed on an ION-TOF IV instrument (IONTOF GmbH, Münster, Germany). Measurements were taken at temperatures of 5 and 40 °C. A pulsed 25-kV Bi_3_^+^ primary ion source was used at a target current of approximately 1 pA to raster two randomly selected 100 × 100 µm areas of the coupon to collect both positive and negative secondary ions. Charge compensation of the samples was accomplished with a pulsed electron flood gun. The mass of secondary ions was determined using a time-of-flight mass analyser. The typical mass resolution (at m/z 41) was just over 6000.

## Results

**Figure 1:**
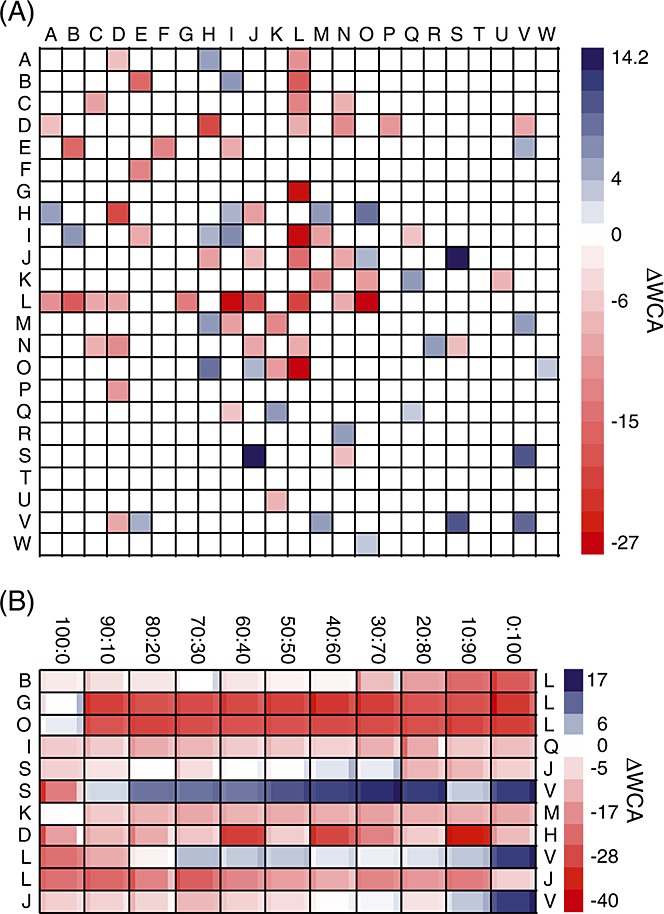
Intensity map showing the change in water contact angle (WCA) when temperature is switched from 8 to 40 °C for (A) the first generation array and (B) the second-generation array. Blue indicates a positive shift, whereas red indicates a negative shift as indicated by the intensity scale. Materials shown in white had a shift in WCA below the LOD (three times the standard deviation of repeated measurements on the pHEMA background). Monomers are indicated by a letter. For (A), monomers were mixed at a 50 : 50 ratio. For (B), the monomer composition ratios are indicated across the top of the figure and denote the ratio between the two monomers indicated as a letter on the left and right of the figure. The large block indicates the value of the change in WCA, whereas the small blocks to the left and right of the large block indicate the mean ± the standard deviation, *n* = 3.

The formation of a first generation array was achieved by printing 279 unique solutions for polymerisation onto a pHEMA-coated glass slide with subsequent UV-initiated curing.^21^ The polymers were formed from 23 amphiphilic monomers (Fig. SI1, Supporting Information). Automated pico litre sessile drop WCA measurements were made for all 279 materials, initially at 8 °C and then at 40 °C, as a screen to identify thermally responsive polymers. This temperature range was chosen because of its biological relevance and the ease at which these temperatures can be achieved in many laboratories. The resultant ΔWCA (WCA^40^ − WCA^8^) for each polymer is shown in Fig. 1(A). The ΔWCA was assumed to be 0 for polymers where the measured ΔWCA was below the limit of detection (LOD) (three times the standard deviation of a measurement). From this initial screen, the top 11 ‘hit’ compositions producing either a positive or negative ΔWCA were selected for a second-generation array where the two monomers from each composition were varied systematically from 0%–100% in increments of 10%. The second-generation array contained a total of 121 polymers, and three replicate arrays were produced on the same slide. The resultant ΔWCA when the temperature was increased from 8 to 40 °C is shown in Fig. 1(B).

**Figure 2:**
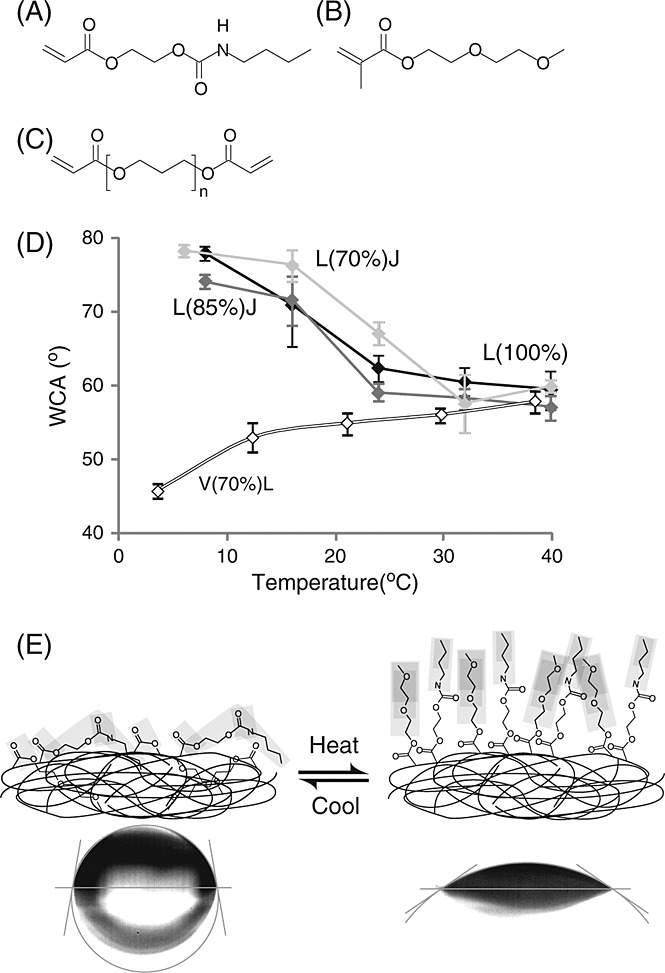
(A–C) The chemical structure of monomers (A) L, (B) J and (C) V. (D) The WCA measured for each of the polymer compositions for temperatures of 8–40 °C. Error bars equal ±one standard deviation, *n* = 9. The monomer compositions studied were L(100%) (♦), L(85%)J(15%) (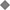), L(70%)J(30%) (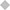) and V(70%)L(30%) (◊). (E) Schematic depiction of the molecular conformation of a copolymer of monomers L and J upon heating or cooling and the corresponding WCA measurements, which used a circle fit. The molecular fragments, which ions enriched at each temperature are likely to have originated from, are highlighted in grey.

The 16 polymer compositions that produced the largest absolute ΔWCA were selected for scale up to 10 mm diameter polymer coupons. The monomer composition of selected ‘hit’ formulations was chosen such that monomer content varied by at least 15% to maximise the compositional variation. The WCA for each of these materials was measured from 8 to 40 °C in increments of 8 °C. The WCA is plotted as a function of temperature in Fig. SI2 (Supporting Information) and for the four materials with the largest overall ΔWCA in Fig. 2(D). A significant difference in the ΔWCA between the measurement on the microarray samples and the polymer coupons was noted for five of the 16 compositions (Fig. SI3, Supporting Information). The different thermo-responsive properties of the polymer coupons could be a result of the decreased surface area : volume ratio, resulting in an altered surface energy. This could cause the material to no longer undergo a temperature-induced change in WCA in the temperature range studied. In summary, the largest negative ΔWCA of −18.5° ± 1.8° was measured for the homopolymer of monomer L [Fig. 2(A)], and the largest positive ΔWCA of 17.1° ± 4.0° was measured for the copolymer V(70%)L(30%). These values are of a similar magnitude to the ΔWCA of 12–23° reported for pNIPAM.^22^–^24^ The inclusion of 2-(2-methoxyethoxy)ethyl methacrylate (J) [Fig. 2(C)] with monomer L did not significantly alter the WCA of the material nor the absolute change in the WCA with temperature but rather increased the temperature at which the WCA of the polymer decreased [Fig. 2(D)].

The four polymer coupons with the largest measured ΔWCA with a change in temperature were analysed by ToF-SIMS at two temperatures to see if any molecular structural changes could be detected at the surface that cause the temperature-induced ΔWCA. It is important to note that these measurements are obtained in ultra high vacuum conditions, and relating them to other environments, for example in aqueous conditions, should be carried out with caution. Nevertheless, any surface enrichment of ions is likely to be indicative of changes that occur at the surface at ambient conditions. A subset of characteristic ions with the largest relative change in intensity when the temperature of the materials was changed between 5 and 37 °C is shown in Table 1 (the corresponding full list of ions is shown in Table SI1). For copolymers L(85%)J(15%) and L(70%)J(30%) and the homopolymer of monomer L, an increase in intensity was observed at low temperature for ions originating from monomer L, such as ions CHNO^−^ and C_8_H_13_NO_3_^−^ and from the acrylate/methacrylate backbone, such as CHO_2_^−^, C_2_H_2_^+^ and CH_3_^+^. These results suggest at 5 °C, the whole monomer L side-group is surface enriched. At high temperature, an increase in intensity was observed for ions originating from ethylene glycol moieties on both monomers L and J, such as C_2_H_5_O_2_^−^, C_3_H_7_O^+^ and C_4_H_5_O_2_^−^ and for ions from the terminus of monomer L, such as C_3_H_7_^+^ and C_2_H_4_N^+^. As these polymers also show a decreased contact angle at 37 °C [Fig. 2(D)], it is likely that with an increased temperature leads to the surface enrichment of hydrophilic groups such as ethylene glycol and di(ethylene glycol). Taken together, these results suggest that the polymer pendant groups are surface enriched at higher temperature, likely because of reduced intramolecular interactions. However at reduced temperature, the monomer backbone is surface enriched as intramolecular interactions dominate and cause rotational movement of the side groups towards the polymer bulk. This is likely caused by the temperature increasing above the polymer's upper critical solution temperature. This conformational change is depicted schematically in Fig. 2(E) and is similar to conformational changes observed on pNIPAM.^25^

**Table 1:** Summary of ion characteristic to each monomer with the highest relative change at the surface of polymer coupons at temperatures of 5 and 37 °C as detected by time-of-flight secondary ion mass spectrometry

L(70%)J(30%)			L(85%)J(15%)			L(100%)			V(70%)L(30%)		
			
Ion	5 °C	37 °C	Ion	5 °C	37 °C	Ion	5 °C	37 °C	Ion	5 °C	37 °C
CHNO^−^	0.00285	0.00194	C_2_H_2_^+^	0.00782	0.00234	C_8_H_13_NO_3_^−^	0.01177	0.00628	C_5_H_10_N^+^	0.00151	0.00083
C_2_H_5_NO_2_^+^	0.00102	0.00070	C_3_H_2_^+^	0.00308	0.00126	C_8_H_11_NO_3_^−^	0.00092	0.00051	C_5_H_9_^+^	0.00281	0.00188
CHO_2_^−^	0.02198	0.01577	C_4_H_2_^+^	0.00216	0.00110	CHO_2_^−^	0.03330	0.02163	C_4_H_7_N^+^	0.00104	0.00071
									C_4_H_7_^+^	0.01251	0.00874
C_2_H_5_NO^+^	0.00145	0.00184	C_4_H_3_NO_2_^−^	0.00005	0.00017	C_2_H_5_NO^+^	0.00122	0.00257	C_7_H_15_NO_2_^+^	0.01063	0.01728
C_2_H_5_O_2_^−^	0.01802	0.02247	C_3_H_7_O^+^	0.00444	0.01457	C_3_H_7_^+^	0.00716	0.01250	C_4_H_9_O^+^	0.00165	0.00259
			C_2_H_5_O_2_^−^	0.01120	0.02794	C_2_H_3_O_2_^+^	0.00369	0.00442	C_3_H_7_NO^+^	0.00345	0.00522
									C_5_H_9_O_2_^+^	0.00187	0.00269

The normalised (total ion count) ion intensities at both temperatures are shown. The top half shows the top ions that decreased with an increase in the temperature, and the bottom half of the table shows ions that increased with increasing temperature.

An increase in WCA was measured for the copolymer of V and L with increasing temperature [Fig. 2(C)], which differs from the other three polymers studied. Analysis by ToF-SIMS revealed an increase in the intensity of characteristic ions C_7_H_15_NO_2_^+^ and C_3_H_7_NO^+^ from monomer L and ions C_4_H_9_O^+^ and C_5_H_9_O_2_^+^ from propylene glycols with increased temperature. With a decreased temperature, the C_4_H_7_^+^ and C_5_H_9_^+^ ions from aliphatic carbon and ions C_5_H_10_N^+^ and C_4_H_7_N^+^ from the terminus of monomer L were found to increase. Monomer V [Fig. 2(C)] is a diacrylate and is, thus, less mobile than monomer L; thus, any conformational changes within this polymer likely result from a rearrangement of monomer L. This suggests that the pendant group of monomer L is surface enriched and possibly upright at lower temperatures, whereas at higher temperature, monomer V is exposed at the surface as monomer L is rotated towards the bulk.

## Conclusion

A high throughput methodology has been demonstrated to identify thermally responsive materials based upon altered hydrophilicity. This approach has been applied to polymer microarrays, resulting in the discovery of novel switchable materials L(100%), L(85%)J(15%), L(70%)J(30%) and V(70%)L(30%) that were scaled up to polymer coupons whilst preserving their stimuli responsive nature. ToF-SIMS analysis provided insight into the molecular conformation changes that cause the temperature-responsive ΔWCA. Specifically, the copolymers of monomers J and L alter between an extended and collapsed surface conformation when the temperature is varied from 5 to 40 °C. This study, which utilised ToF-SIMS with a temperature-controlled stage, represents a novel way to investigate the surface wettability changes of thermo-responsive materials and thus understand their interactions with cells and proteins.
