# Supplementary material for: High throughput discovery of thermo-responsive materials using water contact angle measurements and time-of-flight secondary ion mass spectrometry
Source: Surf Interface Anal. 2012 Mar 8;45(1):181–4. doi: 10.1002/sia.4910 (PMC3579490; doi:10.1002/sia.4910)
Supplement: Supplementary file 1 [file sia0045-0181-SD1.doc]

**High throughput discovery of thermo-responsive materials using water contact angle measurements and time-of-flight secondary ion mass spectrometry**

**– Supplementary information**

Andrew L. Hook^1^, David J. Scurr^1^, Daniel G. Anderson^2^, Robert Langer^2^, Paul Williams^3^, Martyn Davies^1^, Morgan Alexander^1*^

^1^ Laboratory of Biophysics and Surface Analysis, University of Nottingham, Nottingham, NG7 2RD (UK)

^2^ David H. Koch Institute for Integrative Cancer Research, Massachusetts Institute of Technology, 77 Massachusetts Avenue, Cambridge, MA 02139 (USA)

3School of Molecular Medical Sciences, University of Nottingham, Nottingham, NG7 2RD (UK)

* Corresponding author: Email address: [Morgan.Alexander@nottingham.ac.uk](mailto:Morgan.Alexander@nottingham.ac.uk)

Fax number: +44 115 951 5110

1. Chemical structures of monomers used for polymer synthesis.


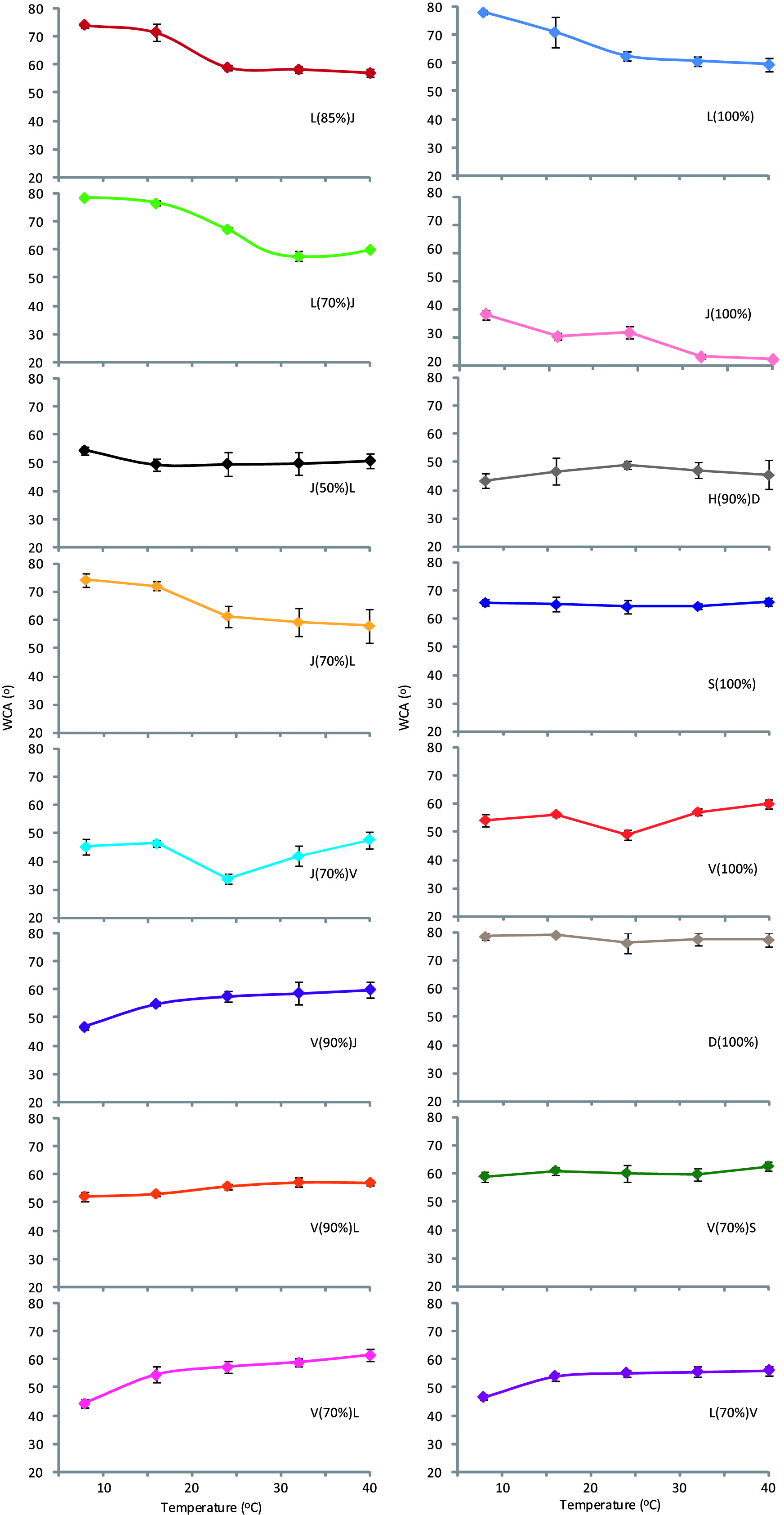


1. WCA versus temperature on scaled up polymer coupons. The composition of each polymer is indicated for each graph. The content of the minor monomer is indicated. The remaining content consists of the secondary monomer given (L(85%)J = 85% of monomer L and 15% of monomer J). The scale bars equal ± 1 standard deviation (n=9).

1. Comparison of the ΔWCA when the sample was heated from 5 °C to 40 °C measured on samples formed on both the microarray format and as coupons. The y=x line is drawn as a guide. The error bars equal ± one standard deviation unit (n=3 for x-error bars; n=9 for y-error bars). A reduced positive ΔWCA was measured for V(100%) (microarray: 15.0 °C ± 1.9 °C; coupon: 5.9 °C ± 1.9 °C) and V(70%)S(30%) (microarray: 16.0 °C ± 1.4 °C; coupon: 3.6 °C ± 1.6 °C) on the polymer coupon compared with the microarray sample. The ΔWCA measured for H(90%)D(10%) decreased significantly from -35.6 °C ± 1.5 °C to 2.1 °C ± 3.8 °C when changed from the microarray to coupon format. Similarly, the ΔWCA measured for J(50%)L(50%) decreased from -14.5 °C ± 1.8 °C to -3.6 °C ± 4.5 °C when changed from the microarray to coupon format.
2. Summary of the ions with the highest relative change at the surface of polymer coupons at temperatures of 5 °C and 37 °C as detected by ToF-SIMS. The normalised (total ion count) ion intensities at both temperatures are shown. The top half shows the top ions that increased with a decrease in the temperature and the bottom half of the table shows ions that decreased with decreasing temperature.

| L(70%)J(30%) | | | L(85%)J(15%) | | | L(100%) | | | V(70%)L(30%) | | |
| --- | --- | --- | --- | --- | --- | --- | --- | --- | --- | --- | --- |
| Ion | 5 °C | 37 °C | Ion | 5 °C | 37 °C | Ion | 5 °C | 37 °C | Ion | 5 °C | 37 °C |
| Na^+^ | 0.00740 | 0.00274 | C_2_H_2_^+^ | 0.00782 | 0.00234 | Na^+^ | 0.02177 | 0.00771 | C_5_H_10_N^+^ | 0.00151 | 0.00083 |
| Cl^-^ | 0.00183 | 0.00103 | C_3_H_2_^+^ | 0.00308 | 0.00126 | Cl^-^ | 0.00145 | 0.00059 | C_5_H_8_N^+^ | 0.00120 | 0.00070 |
| CHNO^-^ | 0.00285 | 0.00194 | C_4_H_2_^+^ | 0.00216 | 0.00110 | C_8_H_13_NO_3_^-^ | 0.01177 | 0.00628 | C_4_H_12_N^+^ | 0.00151 | 0.00098 |
| C_2_H_5_NO_2_^+^ | 0.00102 | 0.00070 | CH_3_^+^ | 0.01090 | 0.00599 | C_8_H_11_NO_3_^-^ | 0.00092 | 0.00051 | C_5_H_9_^+^ | 0.00281 | 0.00188 |
| SO_3_H^-^ | 0.00095 | 0.00068 | C_2_H_3_^+^ | 0.05082 | 0.02841 | SO_3_^-^ | 0.00563 | 0.00331 | C_4_H_7_N^+^ | 0.00104 | 0.00071 |
| CHO_2_^-^ | 0.02198 | 0.01577 | C_3_H_3_^+^ | 0.03519 | 0.01978 | C_4_H_3_O^+^ | 0.00189 | 0.00116 | C_4_H_7_^+^ | 0.01251 | 0.00874 |
| SO_3-_ | 0.00281 | 0.00204 | C^-^ | 0.01387 | 0.00787 | SO_3_H^-^ | 0.00160 | 0.00103 | C_4_H_8_N^+^ | 0.00454 | 0.00324 |
|  |  |  | O^-^ | 0.07937 | 0.04562 | CHO_2_^-^ | 0.03330 | 0.02163 | C_6_H_9_^+^ | 0.00290 | 0.00209 |
| C_2_H_5_NO^+^ | 0.00145 | 0.00184 | Na^+^ | 0.00616 | 0.03273 | C_2_H_5_NO^+^ | 0.00122 | 0.00257 | C_7_H_15_NO_2_^+^ | 0.01063 | 0.01728 |
| C_2_H_5_O_2_^-^ | 0.01802 | 0.02247 | C_4_H_3_NO_2_^-^ | 0.00005 | 0.00017 | C_3_H_7_^+^ | 0.00716 | 0.01250 | Cl^-^ | 0.00120 | 0.00191 |
| C_3_H_7_^+^ | 0.00833 | 0.01017 | C_3_H_7_O^+^ | 0.00444 | 0.01457 | C_4_H_9_^+^ | 0.00271 | 0.00387 | C_4_H_9_O^+^ | 0.00165 | 0.00259 |
| C_2_H_3_O_2_^+^ | 0.00345 | 0.00408 | C_6_H_13_O_3_^-^ | 0.00046 | 0.00141 | CHNO^-^ | 0.00140 | 0.00197 | C_3_H_7_NO^+^ | 0.00345 | 0.00522 |
| C_3_H_4_O^-^ | 0.00103 | 0.00120 | CH_3_O^-^ | 0.00087 | 0.00250 | C_3_H_5_^+^ | 0.09758 | 0.12462 | C_5_H_9_O_2_^+^ | 0.00187 | 0.00269 |
| C_5_H_10_NO_2_^-^ | 0.01434 | 0.01676 | C_4_H_5_O_2_^-^ | 0.00258 | 0.00671 | C_3_H_5_^+^ | 0.09639 | 0.12229 | Na^+^ | 0.00363 | 0.00518 |
| C_4_H_5_O_2_^-^ | 0.01549 | 0.01795 | C_2_H_5_O_2_^-^ | 0.01120 | 0.02794 | C_2_H_3_O_2_^+^ | 0.00369 | 0.00442 | C_4_H_9_O_3_^+^ | 0.00275 | 0.00382 |
| C_3_H_6_^+^ | 0.02708 | 0.03118 | C_2_H_3_^-^ | 0.00120 | 0.00285 | C_3_H_7_O^+^ | 0.15528 | 0.17959 | C_4_H_7_NO_2_^+^ | 0.00228 | 0.00314 |
| C_3_H_5_^+^ | 0.08422 | 0.09628 | C_3_H_7_O_2_^-^ | 0.00046 | 0.00109 | C_2_H_5_O_2_^-^ | 0.00551 | 0.00630 | C_6_H_11_O_2_^+^ | 0.00109 | 0.00147 |
| C_2_H_2_O_2_^-^ | 0.00872 | 0.00997 | C_3_H_3_O^-^ | 0.00156 | 0.00368 | C_2_H_4_N^+^ | 0.00276 | 0.00306 |  |  |  |
|  |  |  |  |  |  | C_3_H_6_^+^ | 0.03807 | 0.04185 |  |  |  |
|  |  |  |  |  |  | CH_3_O^-^ | 0.00228 | 0.00247 |  |  |  |
